# Coulomb-corrected molecular orbital tomography of nitrogen

**DOI:** 10.1038/srep23236

**Published:** 2016-03-22

**Authors:** Chunyang Zhai, Lixin He, Pengfei Lan, Xiaosong Zhu, Yang Li, Feng Wang, Wenjing Shi, Qingbin Zhang, Peixiang Lu

**Affiliations:** 1 School of Physics and Wuhan National Laboratory for Optoelectronics, Huazhong University of Science and Technology, Wuhan 430074, China; 2 Laboratory of Optical Information Technology, Wuhan Institute of Technology, Wuhan 430205, China

## Abstract

High-order harmonic generation (HHG) from aligned molecules has provided a promising way to probe the molecular orbital with an Ångström resolution. This method, usually called molecular orbital tomography (MOT) replies on a simple assumption of the plane-wave approximation (PW), which has long been questioned due to that PW approximation is known to be valid in the keV energy region. However, the photon energy is usually no more than 100 eV in HHG. In this work, we experimentally reconstruct the highest occupied molecular orbital (HOMO) of nitrogen (N_2_) by using a Coulomb-corrected MOT (CCMOT) method. In our scheme, the molecular continuum states are described by a Coulomb wave function instead of the PW approximation. With CCMOT, the reconstructed orbital is demonstrated to agree well with the theoretical prediction and retain the main features of the HOMO of N_2_. Compared to the PW approximation method, the CCMOT shows a significant improvement in eliminating the artificial structures caused by PW approximation.

High-order harmonics can be generated when atoms, molecules are irradiated by an intense laser pulse. These harmonics span a very broad spectral range and are locked in phase, leading to the generation of coherent attosecond pulses, which have made a breakthrough of time-resolved measurements in the attosecond time-scale^1^,^2^. On the other hand, the broadband spectra of HHG involve rich information of the atomic and molecular targets and provides a promising way to detect the target structure and dynamics^3^,^4^,^5^,^6^. The HHG process is well explained by the three-step model^7^: the most active electron of the atom or molecules is firstly freed and secondly is accelerated by the intense laser field. Finally, the electron returns to the parent ion with emitting high-order harmonics when the driving field changes its direction. A quantum theory following this idea has also been developed within the strong-field approximation (SFA)^8^. According to this theory, in the third step, the accelerated electron is scattered by the parent ion in a well-defined direction. This makes HHG of molecules dependent strongly on the molecular orientation. By performing a tomographic analysis of the harmonic signals obtained at different molecular orientations, an image of the orbital from which the electron was released can be reconstructed. This idea was first proposed and demonstrated by Itatani *et al.*^9^ to reconstruct the highest occupied molecular orbital (HOMO) of N_2_. Since then, molecular orbital tomography (MOT) with HHG has attracted a great deal of attention for its potential application to monitor the dynamics of chemical reactions^10^,^11^,^12^,^13^ and recently it has been applied to some other species such as CO_2_^14^ and asymmetric molecule CO^15^,^16^.

The MOT procedure relies on the basic assumption of strong-field approximation (SFA)^8^ theory: the electron dynamics in the second step is determined by the laser field with no influence of the parent ion. With this assumption, the continuum electron wave functions are approximated by the plane waves (PWs)^9^. Then transition dipole is given in the form of Fourier transform of the HOMO weighted by the dipole operator and the molecular orbital can be directly reconstructed by performing inverse Fourier transform of the dipole moment. However, it’s well known that the PW approximation is a priori inadequate in the range of relatively low electron kinetic energies from several tens to 100 eV where the HHG experiments are usually performed. Recent works have demonstrated that the Coulomb potential of the parent ion could distort the continuum wave function and further influence harmonic emission from molecules^17^,^18^. In particular, Walters *et al.*^19^ have pointed out that the accuracy of MOT can be greatly affected by the distortion of scattering wave functions due to the electron-ion interaction. For these reasons, the MOT based on PW approximation has raised a long-standing controversy^20^,^21^ about its applicability to real-world molecular systems since it was proposed.

To overcome the defect of PW approximation, much effort has been expended to correct the SFA by including Coulomb effects in strong-field ionization^22^,^23^. For MOT, it’s also desirable to take the Coulomb effects of the parent ion on the continuum electrons into account. In this work, we demonstrate a Coulomb-corrected MOT (CCMOT) method to reconstruct the HOMO of N_2_ in experiment. By using a two-center Coulomb wave to describe the molecular continuum states, we successfully reconstruct the HOMO of N_2_. The reconstructed orbital matches well with the theoretical result and retains the main features of the Hartree-Fock orbital. Compared to the reconstruction based on PW approximation, the CCMOT can effectively remove the additional structures introduced by PW approximation in the reconstructed orbital. Our result shows a significant improvement over the PW result and provides a more accurate method for MOT.

## Results

**Figure 1:**
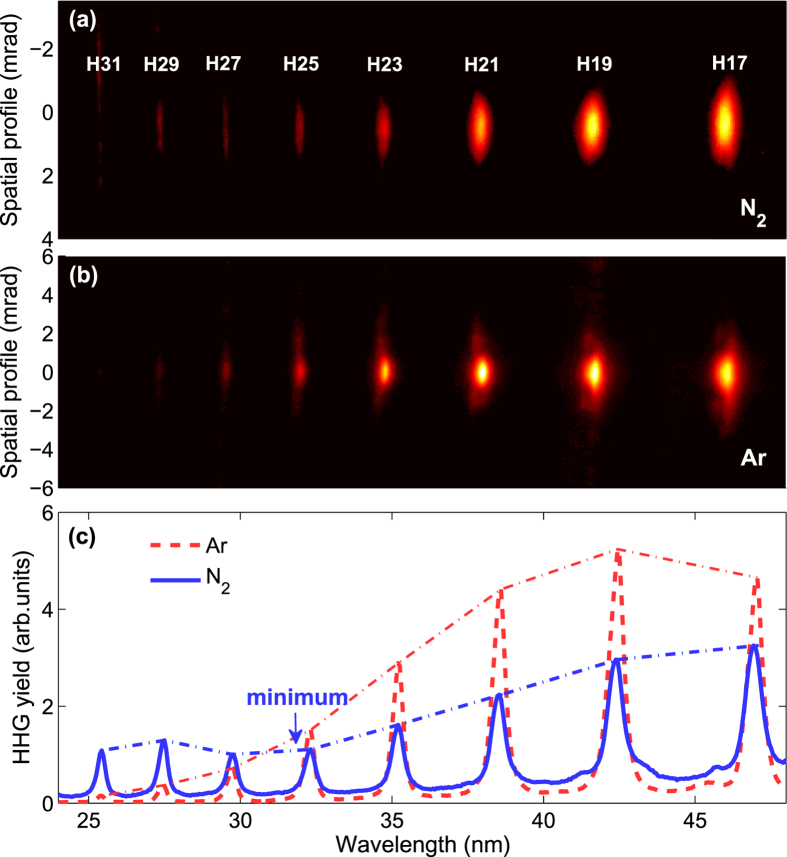
Measured harmonic spectra from (**a**) randomly aligned (without pump pulse) N_2_ molecules and (**b**) the reference atomic gas Ar. (**c**) The spatially integrated HHG signals for the spectra in (**a**) (solid line) and (**b**) (dashed line).

In our experiment, we have measured the harmonic emission from N_2_ (*I*_*p*_ = 15.58 eV) and its reference atom Ar (*I*_*p*_ = 15.76 eV) with the same laser conditions (see Methods). Figure 1(a,b) display the spatially resolved harmonic spectra generated from isotropically aligned (without pump pulse) N_2_ and Ar respectively and their corresponding spatially integrated HHG signals are presented as the solid (N_2_) and dashed (Ar) lines in Fig. 1(c). One can see that, with the increase of harmonic order, the HHG yield from Ar (dashed line) decreases rapidly. While for N_2_, it presents a minimum at 25th harmonic in the harmonic spectrum.

**Figure 2:**
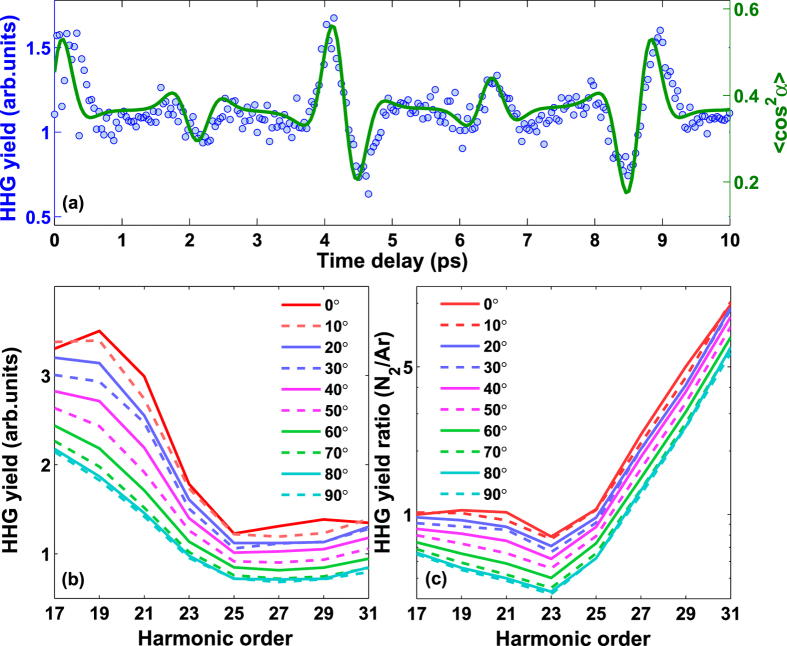
(**a**) Normalized HHG yield of the 17th harmonic (circles) as a function of the time delay between the pump and probe pulses. The green curve displays the calculated temporal evolution of the corresponding alignment degree 〈cos^2^*α*〉. Here, the pump and probe pulses have parallel polarizations. (**b**) Measured harmonic spectra at *τ* = 4.1 ps with the alignment angle rotated from 0° to 90° with a step of 10°. (**c**) Same to (**b**), but the harmonic strength is normalized to that of the reference atom Ar.

To achieve the MOT, one needs to align the molecules in the laboratory frame. In our experiment, this is performed by using a pump pulse with its polarization rotated by a half-wave plate, and the harmonic generation is driven by a probe pulse that has variable time delay with respect to the pump pulse (see Methods). In Fig. 2(a), we present the measured HHG yield (circles) of the 17th harmonic from aligned N_2_ as a function of the pump-probe delay (*τ*). Here, the pump pulse has parallel polarization with respect to the probe pulse, and the HHG yield has been normalized to that measured without pump pulse (isotropic alignment). The HHG yield is significantly modulated with a period given by the rotational period of N_2_ (*T*_*rot*_ = 8.4 ps). The calculated evolution of the alignment degree 〈cos^2^*α*〉 (calculation details can be found in previous reports^24^,^25^), where *α* is the angle between the molecule axis and the pump polarization, is also displayed as the solid line. The temporal evolution of HHG yield is in good agreement with that of 〈cos^2^*α*〉. At *τ* = 4.1 ps, where the 〈cos^2^*α*〉 reaches the maximum, we have measured the HHG signals with alignment angle (the angle between the pump and probe polarization directions) rotated from 0° to 90° with a step of 10°. Corresponding results are presented in Fig. 2(b). The strength of each harmonic is demonstrated to decrease monotonously as the alignment angle increases. This result is consistent with the previous experiments^9^,^10^. Figure 2(c) shows the harmonic yield ratios between N_2_ and Ar (*i.e.*, 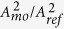 with *A*_*mo*_ and *A*_*ref*_ being the amplitude of the HHG of molecules and reference atoms.) at different alignment angles. In the range from 25th to 31st harmonic, the ratio increases rapidly due to the decrease of the HHG yield from Ar. To ensure the spatial quality of the orbital reconstruction, the experimental data has been further extrapolated up to 360° by imposing the assumed symmetry of the HOMO of N_2_. To determine the recombination dipole *d*_*mo*_ (**k**), the harmonic phases (*ϕ*_*mo*_, *ϕ*_*ref*_) are also required. In this work, we utilize both the experimental and theoretical phases to reconstruct the molecule orbital. The experimental phases (EPs) are obtained from Haessler’s experiment^10^. The theoretical phases (TPs) are calculated by the quantitative rescattering theory (QRS)^21^ with the same parameters as in experiment.

**Figure 3:**
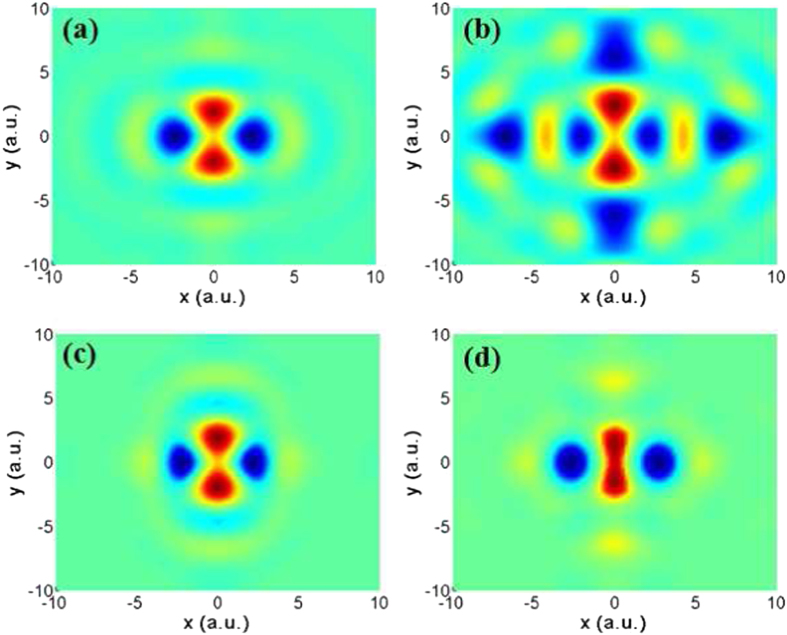
Reconstructed molecular orbital of N_2_: (**a**) the HF orbital filtered as in the experimental conditions, (**b**) the PW reconstruction by using the previous experimental data^10^, (**c**) reconstructed using TCC+TP, and (**d**) reconstructed using TCC+EP.

With the recombination dipole determined, we next perform the molecule orbital reconstruction. In Fig. 3, we show the HOMO images of N_2_ reconstructed with the CCMOT (see Methods). Figure 3(c,d) are the results reconstructed by using the TCC wave in combination with the theoretical (TCC+TP) and experimental (TCC+EP) phases, respectively. Here, we must emphasize that the harmonic bandwidth in our experiment is limited from 17th to 31st harmonics. Therefore for comparison, we have calculated the Hartree-Fock (HF) orbital filtered for experimental samplings as a benchmark in Fig. 3(a). To obtain the filtered HF orbital, we first calculate the recombination dipole *d*_*HF*_ (**k**) by using the HF HOMO [*ψ*_*HF*_ (**r**)] in terms of 
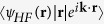
, then the dipole *d*_*HF*_ (**k**) is filtered for the experimental harmonics (17th–31st harmonics) and orientation (0°–90°) samplings, and projected along the driving laser polarization direction. Finally the filtered HF orbital is obtained by performing the inverse Fourier transform of the filtered dipole. One can see that, our experimental reconstructions (both the cases of TCC+TP and TCC+EP) agree well with the simulated one, which exhibits alternating positive and negative lobes and two nodal planes along the y direction. Due to the restricted harmonic bandwidth, the spatial frequency of the orbital is filtered and therefore the reconstructed orbital is elongated. In Fig. 3(b), it shows the PW result reconstructed by using the experimental data in ref. 10. Compared to the simulated orbital, the reconstruction based on the PW approximation introduces some artificial structures in the spatial range of |*x*|, |*y*| > 5 a.u. These structures can be effectively eliminated by considering the Coulomb effects as shown in Fig. 3(c,d). Note that our PW result in Fig. 3(b) and Haessler’s result^10^ are different from that by Itatani *et al.*^9^, especially in the spatial range of |*x*|, |*y*| > 5 a.u., which is due to the difference in the samplings of harmonic range. Moreover, by using the CCMOT method, we also successfully reproduce the orbitals of other non-polar molecules, e.g., O_2_ and CO_2_. The CCMOT can also be extended to heteronuclear molecules (e.g., CO) in combination with other techniques that can decouple the HHG with the recollision from the head or end of the molecule^15^,^16^,^26^, such as decoding odd-even harmonics^15^ or using two-color method^16^.

## Discussion

**Figure 4:**
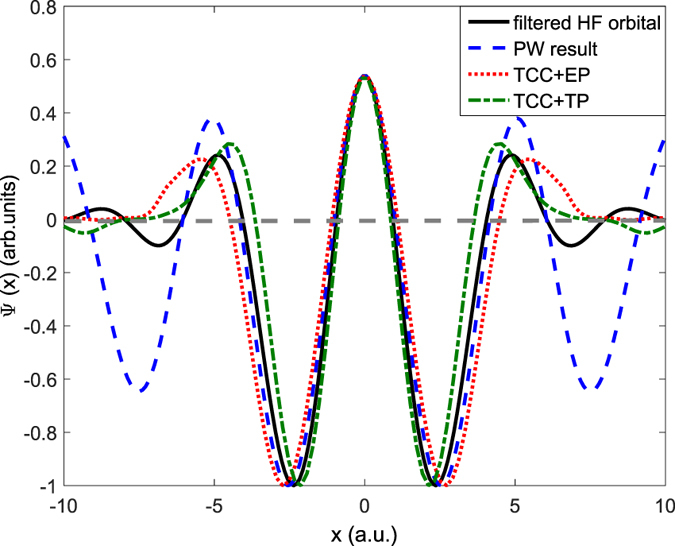
Slices along the internuclear axis for the reconstructed molecular orbitals of N_2_ in Fig. 3. The black solid, blue dashed, green dash-dotted, and red dotted lines are for cases of theoretical filtered HF orbital [Fig. 3(a)], PW result reproduced by using the experimental data^10^ [Fig. 3(b)], TCC+TP [Fig. 3(c)], and TCC+EP [Fig. 3(d)], respectively.

For a quantitative comparison, we have also plotted the slices (along the internuclear axis) of the above four reconstructed orbitals in Fig. 4. It’s obvious that the two line profiles based on the CCMOT method [*i.e.*, TCC+TP (green dash-dotted line) and TCC+EP (red dotted line)] are essentially consistent with the theoretical result (black solid line). Besides, the internuclear distances (defined as the distance between the nodes of the lobes along the molecular axis) reconstructed by TCC+TP and TCC+EP are 1.92 a.u. and 2.02 a.u., which are both very close to the exact value of 2.06 a.u. For the case of PW approximation (blue dashed line), the profile line agrees well with the simulated result in spatial range of |*x*| < 5 a.u. Beyond this range, it shows deep modulations, which arise from the additional structures in Fig. 3(b) that do not exist in the exact HOMO image. In contrast, the CCMOT provides a more accurate reproduction of the molecule orbital.

In summary, we have experimentally demonstrated the molecule orbital reconstruction by using a CCMOT method. With this method, the molecule orbital can be reproduced from the continuum wave functions without the PW approximation. By employing the two-center Coulomb wave which includes the main Coulomb effects to describe the continuum states, we successfully reproduce the HOMO of N_2_. The reconstructed orbital retains the main features of the Hartree-Fock orbital and shows good agreement with the theoretical result. Compared to PW result, the reconstruction with Coulomb corrections effectively eliminates the artificial structures induced by the PW approximation. Our result provides a more accurate method for molecule orbital reconstruction and is conducive to clarify the long-standing controversy in the original MOT theory.

## Methods

### Experimental methods

**Figure 5:**
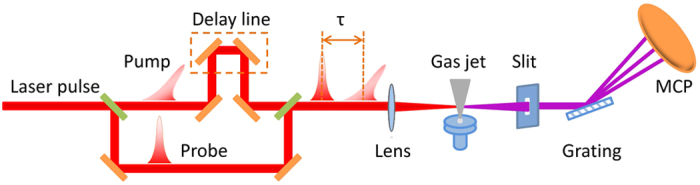
Schematic layout of the experiment.

Our experiment is preformed by using a commercial Ti:sapphire laser system (Legend Elite-Duo, Coherent, Inc.), which delivers 30-fs, 800-nm pulses at a repetition rate of 1 kHz. Figure 5 shows a schematic layout of the experiment. The output laser is spilt into two beams. One is extended to 50-fs for aligning the molecules (pump pulse) and the other is for generating harmonics (probe pulse). The polarization of the pump pulse is rotated by a half-wave plate. Iris diaphragms is used to independently adjust the laser beam size of the pump and probe pulses. The delay between the pump and probe pulses can be changed by the delay line. The two beams are collinearly focused on a pulsed supersonic gas jet emitted from a 100-*μ*m diameter nozzle. The stagnation pressure of the gases is maintained at 2 bars and the gas jet is placed 2 mm after the laser focus. The temperature of the gas in the interaction region is estimated to be about 70 K^27^,^28^, which ensures a high degree of molecular alignment. Throughout our experiment, the laser energies of the pump and probe pulses are kept constant and the corresponding intensities are estimated to be 5 × 10^13^ W/cm^2^ and 2 × 10^14^ W/cm^2^, respectively. The generated high-order harmonic spectrum is detected by a home-made flat-field soft x-ray spectrometer^29^.

### Theoretical methods

According to the SFA theory, the induced dipole moment for HHG can be given as a factorized expression^9^,^10^,^12^:


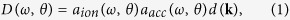


where *θ* is the angle between the driving-field polarization and the molecular axis. The first factor *a*_*ion*_(*ω*, *θ*) represents the tunnel ionization amplitude, *a*_*acc*_(*ω*, *θ*) describes the propagation amplitude of the recolliding electron wave packet in the continuum, and *d* (**k**) is the recombination matrix element between the initial orbital and the continuum wave function. In experiment, to extract the recombination dipole *d* (**k**) from the measured harmonic signals, one should determine the first two factors. This can be achieved by measuring the harmonic emission from a reference atom that has the same ionization potential (*I*_*p*_) with the molecule. By measuring the amplitude *A*(*ω*, *θ*) and phase *ϕ*(*ω*, *θ*) of the generated harmonics, the dipole moment *D*(*ω*, *θ*) can be determined as: *D* (*ω*, *θ*) = *A*(*ω*, *θ*)*e*^*iϕ*(*ω*, *θ*)^. The recombination dipole matrix element *d*_*ref*_ (**k**) of the reference atom can be known from theory, then the molecular dipole can be obtained by:


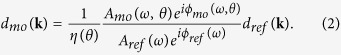


Here, *A*_*mo*_, *A*_*ref*_, *ϕ*_*mo*_, and *ϕ*_*ref*_ are the amplitude and phase of the harmonics generated from molecule and reference atom, respectively. The harmonic amplitudes *A*_*mo*_, *A*_*ref*_ can be directly obtained from the measured harmonic yields (i.e., 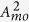 and 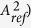 of the N_2_ molecule and Ar atom. In our reconstruction, both the experimental and theoretical phases have been used. The experimental phases (EPs) are obtained from Haessler’s experiment^10^. The theoretical phases (TPs) are calculated by the quantitative rescattering theory (QRS)^21^ with the same parameters as in experiment. The scaling factor *η*(*θ*) stands for the *θ*-dependent tunnel ionization amplitude in the molecule^11^,^12^, which is calculated according to the molecular ADK model^30^. For a multi-electron system, the recombination dipole is given by^11^,^12^


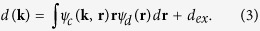


where *ψ*_*d*_ is the Dyson orbital, *d*_*ex*_ is the ‘exchange correction’ term, which contains contributions by all other bound orbitals of the neutral and becomes important only if orbital relaxation upon ionization is significant^11^. Under the Koopmans’ approximation^11^, such a relaxation is negligible and the exchange term *d*_*ex*_ will vanish. Also, the Dyson orbital *ψ*_*d*_ will degenerate to the simple HF orbital *ψ*_0_, and the multi-electron recombination dipole [Eq. (3)] will become the single-active-electron dipole,


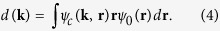


Recall that under the PW approximation, the continuum wave function *ψ*_*c*_ is in the form of 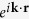. The ground-state wave function can be directly reconstructed by performing inverse Fourier transform of 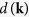. In our work, we leave out the PW approximation and adopt the two-center Coulomb (TCC) wave, which is the solution of the two-body Coulomb continuum problem that includes the main Coulomb effects on the recolliding wave packet^31^, to describe the continuum states. Then, the direct Fourier transform is inoperative. To retrieve the orbital wave function, we first expand the TCC wave in the momentum space by performing the transformation 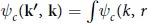, and record the transformation matrix *S* with 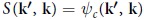
31. In general, this matrix is not diagonal for the reason that the continuum states will have the nonzero component at 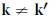 due to the distortions by the molecular potential. By replacing the 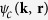 in equation (3) with 
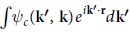
, the equation (3) can be written as


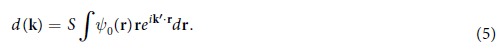


Then the orbital wave function can be reconstructed by *ψ*_0_(**r**) = FT^−1^ [*S*^−1^*d*(**k**)]/**r**.

## Additional Information

**How to cite this article**: Zhai, C. *et al.* Coulomb-corrected molecular orbital tomography of nitrogen. *Sci. Rep.* **6**, 23236; doi: 10.1038/srep23236 (2016).
